# Supplementary material for: Integration of Expression QTLs with fine mapping via SuSiE
Source: medRxiv. 2023 Oct 6:2023.10.03.23294486. Preprint. [Version 2] doi: 10.1101/2023.10.03.23294486 (PMC10593033; doi:10.1101/2023.10.03.23294486)
Supplement: Supplement 1 [file NIHPP2023.10.03.23294486v2-supplement-1.pdf]

medRxiv preprint doi: https://doi.org/10.1101/2023.10.03.23294486; this version posted October 6, 2023. The copyright holder for this preprint (which was not certified by peer review) is the author/funder, who has granted medRxiv a license to display the preprint in perpetuity.
It is made available under a CC-BY 4.0 International license .

Supporting information

S1 Algorithm. Iterative Bayesian stepwise selection (IBSS) algorithm [8].

medRxiv preprint doi: https://doi.org/10.1101/2023.10.03.23294486; this version posted October 6, 2023. The copyright holder for this preprint (which was not certified by peer review) is the author/funder, who has granted medRxiv a license to display the preprint in perpetuity.
It is made available under a CC-BY 4.0 International license .

Algorithm 1 IBSS

Require: data $\mathbf{X}, \mathbf{y}$, number of effects K, hyperparameters $\sigma^2, \boldsymbol{\sigma_0^2}$

1: Initialize posterior means $\bar{\mathbf{b}}_\mathbf{k} = 0,\ k = 1, .., K$
2: **repeat**
3: **for** `k in 1,...,K` **do**
4: $\bar{\mathbf{r}}_\mathbf{k} \leftarrow \mathbf{y} - \mathbf{X} \sum_{k' \neq k} \bar{\mathbf{b}}_{\mathbf{k'}}$ ▷ expected residuals without kth single effect
5: $(\boldsymbol{\alpha_k}, \boldsymbol{\mu_{1k}}, \boldsymbol{\sigma_k^2}) \leftarrow SER(\mathbf{X}, \bar{\mathbf{r}}_\mathbf{k}, \sigma^2, \sigma_{0k}^2)$
6: $\bar{\mathbf{b}}_\mathbf{k} \leftarrow \boldsymbol{\alpha_k} \cdot \boldsymbol{\mu_{1k}}$ ▷ $\cdot$ denotes elementwise multiplication
7: **end for**
8: **until** convergence **return** $\boldsymbol{\alpha_k}, \boldsymbol{\mu_{1k}}, \boldsymbol{\sigma_k^2}$

S1 Table. Summary information of AD mediators. We summarize the chromosome, SNP ID, AD risk gene, indicator of the coding region, whether or not this mediator can be identified by SuSiE and SuSiE^2, and the estimated PIPs for every AD mediator in this table.

Chromosome	SNP ID	Gene	Region	SuSiE	SuSiE^2	PIP(SuSiE)	PIP(SuSiE^2)
1	rs4575098	ADAMTS4	non-coding	FALSE	FALSE	0.17953	0.18719
2	rs13025717	BIN1	non-coding	FALSE	FALSE	0.01919	0.02976
6	rs1004173	TNFRSF21	non-coding	TRUE	TRUE	0.10046	0.10046
7	rs6464547	TMEM139	non-coding	FALSE	FALSE	0.00743	0.00743
10	rs7920721	USP6NL	non-coding	FALSE	FALSE	0.00006	0.00020
10	rs7900536	TSPAN14	non-coding	FALSE	TRUE	0.11388	0.13001
11	rs2276412	SORL1	coding	FALSE	FALSE	0.00612	0.00263
11	rs3740688	SPI1	coding	TRUE	TRUE	0.49861	0.51323
11	rs1237999	PICALM	non-coding	FALSE	TRUE	0.04728	0.14347
14	rs3829409	C14orf93	coding	FALSE	FALSE	0.03634	0.03447
14	rs10130373	RIN3	non-coding	TRUE	TRUE	0.02232	0.02281
15	rs2289702	CTSH	coding	FALSE	TRUE	0.02941	0.05366
15	rs653765	ADAM10	non-coding	FALSE	FALSE	0.00085	0.00048
15	rs72749561	MEX3B	non-coding	FALSE	FALSE	0.00368	0.00361
17	rs3816913	USP6	coding	FALSE	FALSE	0.02734	0.03049
17	rs28618326	NGFR	non-coding	TRUE	TRUE	0.05928	0.05928
19	rs3764645	ABCA7	coding	FALSE	FALSE	0.01769	0.02767
19	rs12459419	CD33	coding	TRUE	TRUE	0.43724	0.43503
19	rs2303696	REX1BD	non-coding	FALSE	TRUE	0.01170	0.02585
20	rs17462136	CASS4	non-coding	FALSE	FALSE	0.00798	0.00777

The SuSiE column represents whether or not the original SuSiE can identify the corresponding AD mediator. The SuSiE^2 column is similar.
